# Novel High-Sensitivity Racetrack Surface Plasmon Resonance Sensor Modified by Graphene

**DOI:** 10.3390/molecules23071726

**Published:** 2018-07-14

**Authors:** Jun Zhu, Zhengjie Xu, Yuanmin Huang

**Affiliations:** 1 College of Electronic Engineering, Guangxi Normal University, Guilin 541004, China; xuzhengjie0001@gmail.com; 2 College of Mathematics and Statistic, Guangxi Normal University, Guilin, 541004, China; huangyuanmin@mailbox.gxnu.edu.cn

**Keywords:** surface plasmon resonance, tunable device, modified graphene, refractive index, temperature detector

## Abstract

In order to overcome the existing challenges presented by conventional sensors, including their large size, a complicated preparation process, and difficulties filling the sensing media, a novel high-sensitivity plasmonic resonator sensor which is composed of two graphene-modified straight waveguides, two metallic layers, and a racetrack nanodisk resonator is proposed in this study. The transmission characteristics, which were calculated by the finite element theory, were used to further analyze the sensing properties. The results of quantitative analysis show that the proposed plasmonic sensor generates two resonance peaks for the different incident wavelengths, and both resonance peaks can be tuned by temperature. In addition, after optimizing the structural parameters of the resonator, the Q value and the refractive sensitivity reached 21.5 and 1666.67 nmRIU^−1^, respectively. Compared with other studies, these values translate to a better performance. Furthermore, a temperature sensitivity of 2.33 nm/5 °C was achieved, which allows the sensor to be easily applied to practical detection. The results of this study can broaden the useful range for a nanometer-scale temperature sensor with ultrafast real-time detection and resistance to electromagnetic interference.

## 1. Introduction

Surface plasmon polariton (SPP), allowing the compact storage of optical energy in electron oscillations at the interfaces of metals and dielectrics, has emerged as promising solution to overcome the barrier caused by the diffraction limit of light [1,2,3]. The traditional types of surface plasmon resonance (SPR) sensors are propagating SPR (PSPR) and localized SPR (LSPR) sensors, respectively [4,5]. In recent years, with the continuous development in the plasmonic sensor field, advantages of small size, fast response, and resistance to electromagnetic interference have been sought. This has increased the number of SPR sensor types, such as the refractive index sensor or micro temperature sensor [6,7]. Maier et al. described surface-enhanced spectroscopies and an LSPR refractive index sensor based on metallic ring and disk cavities, obtaining a sensitivity of 1116 nmRIU^−1^ [8]. Based on a planar metamaterial analogue, a plasmonic sensor with 588 nmRIU^−1^ was demonstrated by Giessen et al. [9]. Then, the state-of-the-art design for a refractive sensor was the plasmonic gold mushroom array, and its sensitivity was 1050 nmRIU^−1^ [10]. After that, a sensor based on nanohole arrays, with a sensitivity of 659 nmRIU^−1^, was proposed by Cuevas et al. [11]. A single semiconductor nanowire [12] and a simple metallic nanogroove array [13] were constructed, and their sensitivities reached 235 nmRIU^−1^ and 1200 nmRIU^−1^, respectively. Recently, many research groups have combined photonic devices with graphene materials to establish a new research area called graphene nanophotonics [14]. Furthermore, the self-consistent field approach within the Markovian master equation formalism (SCF-MMEF) was used to further calculate the dielectric function, complex conductivity, and loss function in graphene by F. Karmi [15,16]. They found that propagation length can be higher in nonpolar materials.

However, there are still some room for improvement in the traditional sensor, such as reducing its size, simplifying the preparation process, mitigating the electromagnetic interference, and overcoming the difficultly of filling the sensing media. In this study, we illustrate the design of a novel high-sensitivity plasmonic resonator sensor, which is composed of two graphene-modified straight waveguides, two metallic layers, and a racetrack nanodisk resonator. Furthermore, several problems associated with the traditional sensor can be overcome by the proposed plasmonic resonator sensor, because the SPP has superior capacity for optical nanoscale integration, and the sensing material (organic solution or mixture solution) can very easy fill the racetrack resonator by capillary attraction. 

High Q value and sensitivity, which can make the detection result more intuitive, are critical factors for the plasmonic resonator sensor. In theory, the resonance properties versus incident wavelength for different temperatures can be calculated by a full vector finite element method. After optimization, compared to other studies, the Q value is 5 times higher and the sensitivity is 2–3 orders higher, simultaneously. 

## 2. Sensor Structure and Theoretical Analysis

### 2.1. Sensor Structure

**Figure 1:**
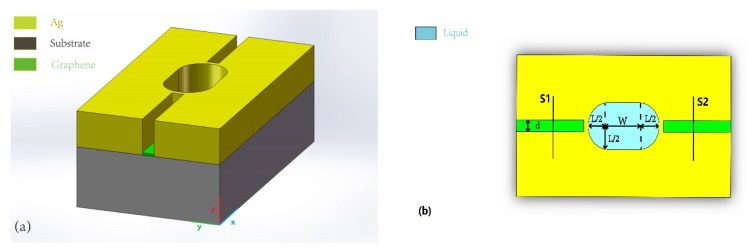
Proposed structure of the racetrack plasmonic resonator sensor. (**a**) 3D schematic diagram of the proposed resonator; (**b**) 2D schematic diagram along *x*–*y* axis.

Figure 1a,b show the proposed plasmonic resonator sensor, consisting of two graphene-modified straight waveguides, two metallic layers, and a racetrack nanodisk resonator. This sensor can lessen the existing challenges that are present in conventional sensors, including their large size, a complicated preparation process, and difficulties in filling the sensing media. To begin, the metallic layer is deposited on the silicon substrate by chemical vapor deposition (CVD). Furthermore, the two straight waveguides and racetrack nanodisk resonator are etched onto the metal layer. Finally, the graphene is deposited to the air area via CVD, and the electronic characteristics of graphene can be adjusted by bias voltage. 

The length and width of the racetrack resonator is set to L and W, respectively. The semicircle on both sides of the resonator has a radius set to L/2, and the width of the straight waveguide is set to d. The gap between the waveguide and racetrack cavity is fixed at 50 nm, and the height of the silver layer is set as 500 nm. S1 and S2 are used to calculate the Poynting vector along the *x* axis.

### 2.2. Theoretical Analysis

It is well known that the dispersion equation of SPP in a metal-insulator-metal structure can be written as [17,18]:

(1) $$\varepsilon_{\mathrm{in}}k_{z2}+\varepsilon_{m}k_{z1}\coth\left(-\frac{{\mathrm{ik}}_{z1}}{2}d\right)=0$$

Because of the low absorption and loss, the two metallic layers are set as Ag, which can improve the propagation length of SPP. In the theoretical calculation, the complex relative permittivity of Ag can be expressed by the Lorentz–Drude model.

Graphene, used in the two straight waveguides, can be seen as a two-dimensional material and is described by: surface conductivity $\sigma_{g}$, which is related to the operation frequency ω, chemical potential $\mu_{c}$, Fermi level $E_{f}$, environmental temperature T, and relaxation time τ. Based on the local random phase approximation (RPA), the graphene conductivity can be calculated by the Kubo formula [19]:

(2) $$\sigma_{g}=\sigma_{intra}\left(\omega ,T,\tau ,\mu_{c}\right)+\sigma_{inter}\left(\omega ,T,\tau ,\mu_{c}\right)$$

The intraband and interband contributions rely on temperature T, angular frequency ω, relaxation time τ, and chemical potential $\mu_{c}$. $k_{B}$ is Boltzmann’s constant, ℏ=h/2π is the reduced Planck’s constant, and –e is the charge of the electron. In our proposed resonator, the chemical potential $\mu_{c}$ is controlled by the application of a gate voltage with suitable chemical doping [20,21]. In our calculation, the thickness of a single graphene sheet is 0.5 nm, the fermi velocity is 9.5 × 10^5^ m/s, relaxation time is 3.59 × 10^−13^ s, and electron mobility is 0.9 m^2^/(V·s).

In order to enhance the sensitivity for temperature-sensing applications, a dielectric material with a high thermo-optic coefficient (dn/dT) value is essential for the racetrack nanodisk resonator. The sensing medium is considered to be a liquid, and its refractive index can be expressed by:

(3) $$n=n_{\mathrm{liquid}}+\left(\mathrm{dn}/\mathrm{dT}\right)\left(T-T0\right)$$

where $\mathrm{dn}/\mathrm{dT}=-4\times 10^{-4}\;\left({{}^{\circ}C}^{-1}\right)$, and $n_{\mathrm{liquid}}$ is the refractive index of the liquid at the reference temperature $T_{0}=25\;{}^{\circ}C$ [22]. Here, we assume $n_{\mathrm{liquid}}=1.35$ from the spectral regime from 500 nm to 2000 nm at 25 °C. Many organic solutions or mixture solutions can be used as sensing media [23]. Here, walnut oil is used as sensing medium. The melting point and boiling point of the media reflect the detection range of sensor. 

The value of the Q-factor is expressed as [24]:

(4) $$Q=f_{\mathrm{res}}/\mathrm{FWHM}$$

FWHM is the full-width at half maximum (FWHM) of the resonance peak, and $f_{\mathrm{res}}$ is the resonance frequency.

## 3. Results and Analysis 

### 3.1. Graphene Properties 

#### 3.1.1. The Conductivity of Graphene

**Figure 2:**
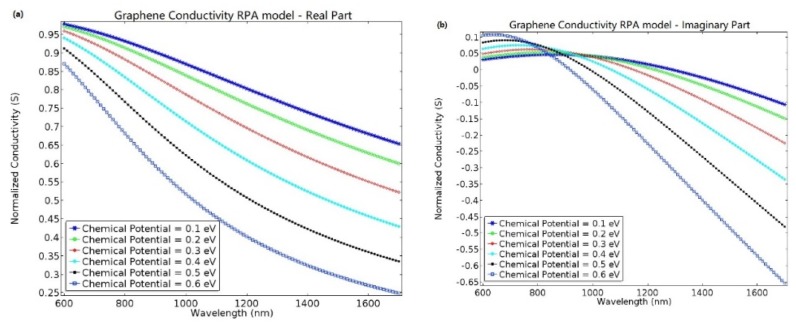
The normalized conductivity of graphene versus wavelength for different chemical potentials, calculated by random phase approximation (RPA) model. The blue asterisk, green circle, red diamond, cyan plus sign, black point, and blue square correspond to chemical potentials of 0.1, 0.2, 0.3, 0.4, 0.5, and 0.6 eV, respectively. (**a**,**b**) are the real part and imaginary part of graphene conductivity, respectively.

Based on the RPA model, the normalized graphene conductivity can be further calculated by the Kubo formula, as shown in Figure 2a,b.

As shown in Figure 2a,b, based upon Equation (2), the relationship between the normalized conductivity of graphene and the wavelength for different chemical potentials can be calculated by the RPA model. As the wavelength increases, both the real part and the imaginary part of the normalized conductivity exhibit the same trend i.e., decrease due to the interaction between interband and intraband electronic transitions. At low frequencies, in the terahertz range, the conductivity of graphene mainly depends on intraband transitions, while, at high frequencies, the interband transition predominates.

#### 3.1.2. Analysis of Transmittance of Plasmonic Resonator Sensor 

Next, the transmittance of the proposed plasmonic resonator sensor, which can be calculated by finite element theory, is defined as: $T=P_{\mathrm{out}}/P_{\mathrm{in}}$, where $P_{\mathrm{in}}=\int PxdS_{1}$, $P_{\mathrm{out}}=\int PxdS_{2}$, and Px is the x component of the Poynting vector.

Firstly, the material on both sides of the straight waveguide is set to air, and the sensing dielectric of the resonator is set as an organic solution or a mixture solution with refractive index 1.6 at zero degrees Celsius and with thermo-optic coefficient $=-4\times 10^{-4}\;\left({{}^{\circ}C}^{-1}\right)$, which is extremely easy to find. The widths of two straight waveguides, d, are set as 50 nm, and the length, L, and width, W, of the proposed plasmonic resonator can be set as 75 nm and 250 nm, respectively. Then, in order to improve the quality factor, the material on both sides of waveguide is modified by graphene for the second analysis. 

**Figure 3:**
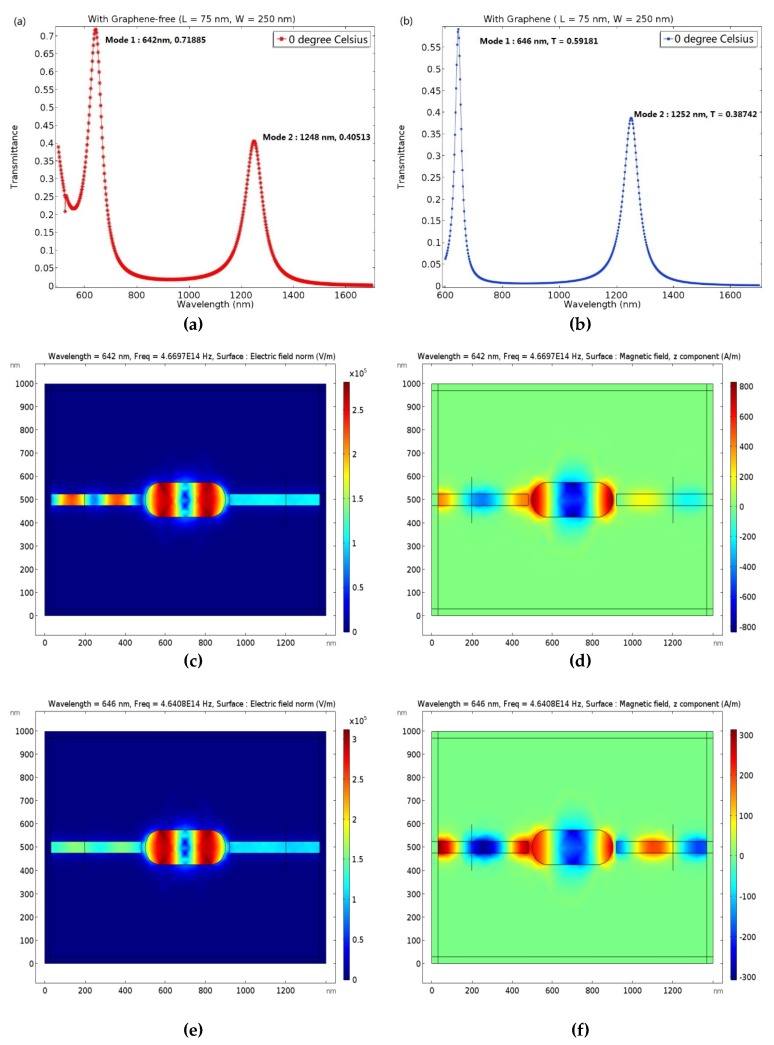
Transmittance of the plasmonic resonator sensor with graphene-free (**a**) and with graphene (**b**) versus different wavelengths at 0 °C. The structural parameters of resonator are L = 75 nm and W = 250 nm, respectively. (**c**) Electric field norm distribution of the plasmonic sensor without graphene at 0 °C and 642 nm. (**d**) Magnetic field distribution of the plasmonic sensor without graphene along the *z* axis at 0 °C and 642 nm. (**e**) Electric field norm distribution of the plasmonic sensor with graphene at 0 °C and 646 nm. (**f**) Magnetic field distribution of the plasmonic sensor with graphene along the *z* axis at 0 °C and 646 nm.

The transmittance and resonant wavelength of the proposed plasmonic resonator sensor without and with graphene are shown in Figure 3a,b, respectively. The electric field norm distribution and the magnetic field along the *z* axis, with and without graphene, can be determined from Figure 3c,f, correspondingly.

Furthermore, as is generally known, when the incident wavelength is satisfied by resonance conduction, the resonance peak occurs. At this time, the transmittance reaches the maximum value. Figure 3a shows that the proposed device without graphene has two resonance peaks at 642 nm and 1248 nm, with increasing incident wavelength. In addition, the transmittance can reach 0.71885 and 0.40513, corresponding to mode 1 and mode 2, respectively. 

As shown in Figure 3b, the two resonance peaks with graphene are excited at 646 nm and 1252 nm, corresponding to mode 1 and mode 2, respectively. The maximum transmittance values of the resonance peaks are 0.59181 and 0.38742, respectively. The resonance phenomenon can be observed more intuitively from the distribution of the electric field norm and magnetic field along the *z* axis, as shown in Figure 3c,f.

Finally, using quantitative analysis, we studied the effects of graphene-free and graphene-modified sensors on the transmittance performance. The resonance peaks both in mode 1 and mode 2 appear to red shift, 4 nm, after the structure is modified by graphene. In addition, the transmittance with graphene is17.7% lower in mode 1 and 4.3% lower in mode 2, respectively, compared to that without graphene. However, when the attention shifts to the visible region, below 800 nm, the minimum values of the proposed plasmonic resonator with graphene and without graphene are 0.05 and 0.2, respectively, a decrease of more than 75%. Because of its outstanding optical characteristics and high carrier mobility, graphene has the capability of enhancing the SPP, which is excited at the interface between Ag and graphene. Furthermore, the graphene waveguide can decrease the propagation loss and provide gain compensation, which is produced by the ohmic loss in the Ag. 

Thus, compared with the graphene-free sensor, the maximum and the minimum values of the proposed resonator modified by graphene decreased by 17.7% and 75%, respectively, which indicates that the graphene has a superior performance in stabilizing the transmittance, especially by decreasing its minimum value. 

#### 3.1.3. The Q-Factor of Plasmonic Resonator

Next, we further quantified the resonance peak by the Q-factor, which can be analyzed using the comprehensive performance of transmittance. By quantifying Figure 3a,b, the FWHM of mode 1 and mode 2 in the device without graphene is 30 nm and 82 nm, respectively, while that with graphene is 30 nm and 68 nm, respectively. Therefore, according to Equation (8), without graphene, we obtain Q-factors of 21.4 and 15.2 for mode 1 and mode 2, respectively, while those with graphene are 21.5 and 18.4, respectively. 

Compared with the graphene-free counterparts, the Q-factors with graphene of mode 1 and mode 2 increase by more than 0.4% and 17.3%, respectively. In addition, compared with 5.4 [12] and 4.188 [24], the highest Q-factor of the proposed plasmonic resonator sensor is 21.5, which is more than 5 times higher than previously reported values. 

### 3.2. Sensing Characteristics

#### 3.2.1. Length Adjustment

**Figure 4:**
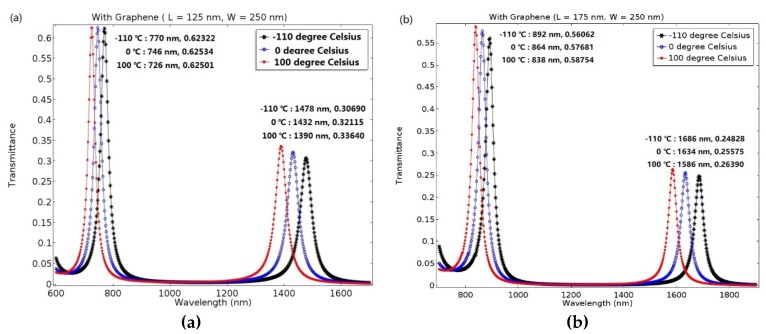
Transmittance of the plasmonic resonator with different structural parameters versus wavelength for −110 °C, 0 °C, and 100 °C, respectively. (**a**) At L = 125 nm, the maximum values for mode 1 and mode 2 are 0.63 and 0.34, respectively. (**b**) At L = 175 nm, the maximum values for mode 1 and mode 2 are 0.58 and 0.26, respectively.

Based on the aforementioned analysis, we believe that the proposed racetrack plasmonic resonator can be applied in a temperature and refractive index sensor. Moreover, the sensing performance can be optimized by adjusting the structural parameters. Firstly, the width of the resonator is fixed at 250 nm, and the length is set to 75 nm and 175 nm, respectively. The transmittance of the proposed device versus wavelength for different temperatures can be seen in Figure 4a,b. 

Figure 4a shows the transmittance of the proposed plasmonic resonator versus varied wavelength for different temperatures at L = 125 nm. The black asterisk, blue circle, and red point correspond to temperatures of −110 °C, 0 °C, and 100 °C, respectively. When the length of the resonator is set to 125 nm, the maximum value of the transmittance is 0.63 and 0.34, corresponding to mode 1 and mode 2, respectively. Furthermore, when the temperature is raised from −110 °C to 100 °C, the resonance peaks of mode 1 and mode 2 shift by 44 nm and 88 nm, respectively. Since the temperature sensitivity is defined as dλ/dT, it results in 748.3 nmRIU^−1^ (1.04 nm/5 °C) and 1496.6 nmRIU^−1^ (2.1 nm/5 °C) for mode 1 and mode 2, respectively. As shown in Figure 4b, when the length is set to 175 nm, the maximum value of transmittance can reach 0.58 and 0.26, corresponding to mode 1 and mode 2 and at this time, and sensitivity of 918 nmRIU^−1^ (1.29 nm/5 °C) and 1700.7 nmRIU^−1^ (2.38 nm/5 °C), respectively, can be achieved. 

Therefore, we can conclude that when increasing the length of the resonator, L, the resonance peak will appear red shifted, which shows great prospects for a tunable device, such as bandpass and band-reject filters. Moreover, when the proposed device works at low temperature, it can eliminate temperature-induced stress and strain. 

**Figure 5:**
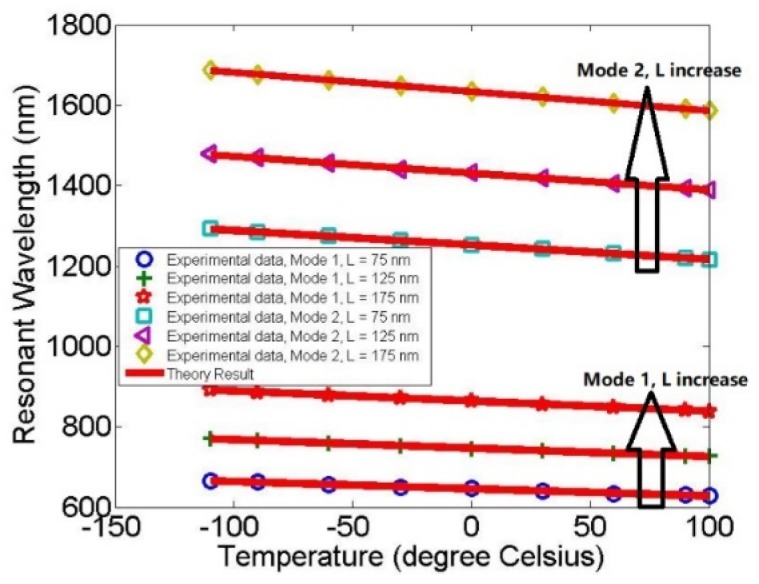
The wavelength at the resonant peak expressed as a function of the temperature for different structural parameters, L. The blue circle, green plus, and red asterisk correspond with L = 75 nm, L = 125 nm, and L = 175 nm, respectively, in mode 1; the cyan square, purple triangle, and yellow diamond correspond with L = 75 nm, L = 125 nm, and L = 175 nm, respectively, in mode 2. The experimental data were calculated by the finite element method and the theoretical result was further validated by MATLAB, corresponding to the red solid line.

We further analyzed the functional relationship between resonance peaks and temperature for different lengths, L, as shown in Figure 5. 

Figure 5 shows the wavelength at the resonant peak as a function of the temperature for different lengths, L. The blue circle, green plus, and red asterisk correspond with L = 75 nm, L = 125 nm, and L = 175 nm, respectively, in mode 1; the cyan square, purple triangle, and yellow diamond correspond with L = 75 nm, L = 125 nm, and L = 175 nm, respectively, in mode 2. In order to perform an accurate analysis, the temperature can be further adjusted from −110 °C to 100 °C, with intervals of 30 °C, and the red solid line is the theoretical calculation.

The results show that the resonance peaks can be tuned by the length of the resonator, L, and the resonance increases with L. At L = 175 nm, W = 250 nm, the maximum value of the sensitivity can reach 918 nmRIU^−1^ (1.29 nm/5 °C) and 1700.7 nmRIU^−1^ (2.38 nm/5 °C) in mode 1 and mode 2, respectively. However, at this time, the maximum value of the transmittance can only reach 0.59 and 0.26, respectively, which still has a room for development. 

Therefore, through comprehensive consideration, we believe that when the length of the resonator is L = 125 nm, the sensitivity of mode 1, 748.3 nmRIU^−1^ (1.04 nm/5 °C), and mode 2, 1496.6 nmRIU^−1^ (2.1 nm/5 °C), is more easily applied to practical application. 

#### 3.2.2. Width Adjustment 

**Figure 6:**
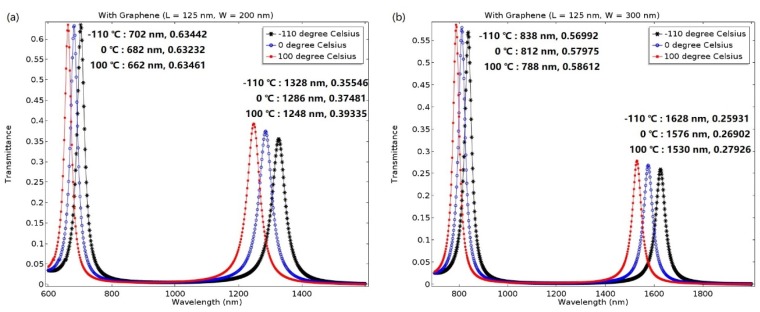
The transmittance of the plasmonic resonator versus different wavelengths for −110 °C, 0 °C, and 100 °C, respectively. (**a**) At W = 200 nm, the maximum value of the transmittance is 0.63 and 0.39 in mode 1 and mode 2, respectively. (**b**) At W = 300 nm, the maximum value of the transmittance is 0.59 and 0.28 in mode 1 and mode 2, respectively.

For this section, based on the optimal parameter of L = 125 nm, we further analyzed the transmittance and sensitivity characteristics by changing the width of resonator, as shown in Figure 6a,b.

The Figure 6a shows the transmittance of the plasmonic resonator versus wavelength for different temperatures. The black asterisk, blue circle, and red point correspond to the temperature at −110 °C, 0 °C, and 100 °C, respectively. When the width of the resonator is set as W = 200 nm, the maximum value of the transmittance is 0.63 and 0.39, respectively, corresponding to mode 1 and mode 2. Moreover, when the temperature is increased from −110 °C to 100 °C, the resonance peaks of mode 1 and mode 2 shift by 40 nm and 80 nm, respectively. Therefore, the sensitivity can reach 680.3 nmRIU^−1^ (0.95 nm/5 °C) and 1360.5 nmRIU^−1^ (1.9 nm/5 °C), respectively. As shown in Figure 6b, the maximum value of the transmittance can reach 0.59 and 0.28, respectively, and the sensitivity is 680.3 nmRIU^−1^ (1.19 nm/5 °C) and 1666.67 nmRIU^−1^ (2.33 nm/5 °C), respectively. In addition, the resonance wavelength will be red shifted with increasing W. 

**Figure 7:**
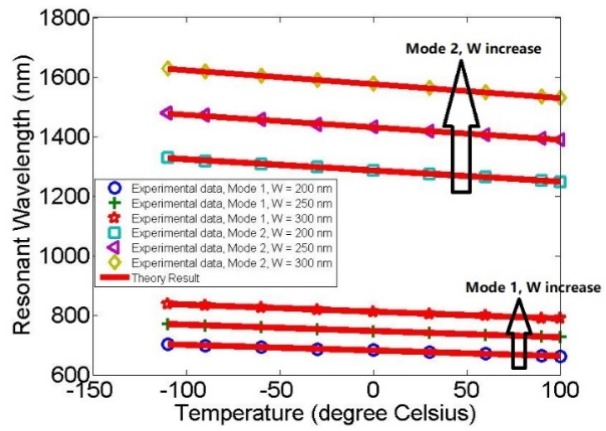
The wavelength at the resonant peak as the function of the temperature for different structural parameters, W. The blue circle, green plus, and red asterisk correspond with W = 200 nm, W = 250 nm, and W = 300 nm, respectively, in mode 1; the cyan square, purple triangle, and yellow diamond correspond with W = 200 nm, W = 250 nm, and W = 300 nm, respectively, in mode 2. The experimental data were calculated by the finite element method and the theoretical result was used further validated by MATLAB, corresponding to the red solid line.

After the aforementioned analysis, in order to accurately analyze the relationship between temperature and resonance wavelength for different structural parameters, the temperature was increased from −110 °C to 100 °C at intervals of 30 °C, and W was adjusted from 200 nm to 300 nm at intervals of 50 nm, as shown in Figure 7.

Finally, the optimal structural parameters of the proposed racetrack plasmonic resonator sensor are L = 125 nm, W = 300 nm, and d = 50 nm. Under these conditions, the sensitivity of the device can reach 1666.67 nm RIU^−1^, which means the temperature sensitivity is 2.33 nm/5 °C. Compared with other studies, as shown in Table 1, it has an outstanding performance as a refractive and temperature sensor.

As shown in Table 1, the proposed device has excellent sensitivity as a refractive sensor and temperature sensor. The sensitivities of the plasmonic gold mushroom arrays [10], nanohole arrays [11], single semiconductor nanowire [12], and a simple metallic nanogroove array [13] are as high as 1015 nmRIU^−1^, 659 nmRIU^−1^, 235 nmRIU^−1^, and 1200 nmRIU^−1^, respectively. However, the proposed plasmonic resonator sensor can achieve a higher sensitivity of 1666.67 nmRIU^−1^ or 2.33 nm/5 °C, which is an improvement of nearly 39.1%, 60.5%, 85.9%, and 28%, correspondingly. 

## 4. Conclusions

A novel high-sensitivity plasmonic resonator sensor, which is composed of two graphene-modified straight waveguides, two metallic layers, and a racetrack nanodisk resonator, is proposed in this study. The transmission characteristics, which were calculated by the finite element theory, were used to further analyze the sensing properties. After that, the proposed device was quantitatively analyzed according to Q-factor and sensitivity. The optimal structural parameters of the proposed racetrack plasmonic resonator sensor are L = 125 nm, W = 300 nm, and d = 50 nm. In addition, based on the optimal parameters, the Q-factor and the sensitivity of the device can reach 1666.67 nmRIU^−1^ (2.33 nm/5 °C) and 21.5, correspondingly, which are much higher than values from other studies. Finally, the resonance peak can be easily tuned by the length and the width of the structure, which can broaden the useful range for a nanometer-scale temperature sensor with ultrafast real-time detection and resistance to electromagnetic interference.

## Figures and Tables

**Table 1:** The sensitivity reported in the literatures.

Sensitivity Reported in Other Studies	
Refs	Sensitivity (nmRIU^−1^)
[6]	0.048 nm/°C
[8]	97–1116
[10]	1015
[11]	659
[12]	235
[13]	1200
[25]	<200
[26]	270
[27]	864–2250
[28]	78
This study	1666.67 or 2.33 nm/5 °C
